# A 10-year-old child presenting with syndromic paucity of bile ducts (Alagille syndrome): a case report

**DOI:** 10.1186/s13256-016-1126-x

**Published:** 2016-11-30

**Authors:** Girish Kumar Pati, Ayaskanta Singh, Preetam Nath, Jimmy Narayan, Pradeep Kumar Padhi, Prasanta Kumar Parida, Kaumudee Pattnaik, Chittaranjan Panda, Shivaram Prasad Singh

**Affiliations:** 1 Department of Gastroenterology, S.C.B. Medical College, Cuttack, Odisha India; 2 Department of Gastroenterology, IMS and Sum Hospital, Bhubaneswar, Odisha India; 3 Department of Pathology, S.C.B. Medical College, Cuttack, Odisha India

**Keywords:** Alagille syndrome, Bile duct paucity, Butterfly vertebra, Café au lait spots, Posterior embryotoxon

## Abstract

**Background:**

Alagille syndrome, a rare genetic disorder with autosomal dominant transmission, manifests with five major features: paucity of interlobular bile ducts, characteristic facies, posterior embryotoxon, vertebral defects, and peripheral pulmonary stenosis. Globally, only 500 cases have so far been reported, with only five cases reported in the Indian subcontinent. Rarely, Alagille syndrome also presents with skin manifestations and early-onset chronic liver disease, which was found in our case. We believe that we report what could be the first case of Alagille syndrome presenting with café au lait spots, as no such published case report could be found in the literature.

**Case presentation:**

We report an unusual case of childhood cholestatic jaundice with neonatal onset of jaundice. A 10-year-old boy from the Indian subcontinent presented with obstructive jaundice from early infancy. He also had recurrent fractures of his upper limb bones, intermittent bleeding from his nose, productive cough, decreased night vision, hyperpigmented spots over his skin, and progressive enlargement of his abdomen. Histological examination of a liver biopsy specimen revealed a paucity of bile ducts and changes suggestive of chronic liver disease. Our patient was diagnosed with Alagille syndrome and managed conservatively but died 1 year after the final diagnosis.

**Conclusions:**

This particular syndromic form of paucity of bile duct disorder has been rarely reported in the Indian literature so far. Our case is notable because the child had café au lait spots and very early onset of chronic liver disease, which is quite rare in Alagille syndrome. We believe this to be the first case report on Alagille syndrome manifesting with café au lait syndrome and such early onset of chronic liver disease.

## Background

Alagille syndrome (AGS) was first described by David Alagille in 1975 as an autosomal dominant disease affecting multiple systems including the liver, heart, eyes, skeleton, and face [1–3]. It is characterized by a paucity of intrahepatic bile ducts with cholestasis and phenotypic manifestations. AGS is most commonly caused by mutations or deletions of the gene encoding Jagged 1 (*JAG1*), a ligand involved in the Notch signaling pathway [4]. We describe the case of a 10-year-old boy who presented with obstructive jaundice and other symptoms related to cholestasis from early infancy. Globally, only 500 cases [1] of AGS have been reported so far, with only five cases in the Indian subcontinent [5]. Rarely, AGS presents with skin manifestations and early-onset chronic liver disease (CLD), which was found in our case and is therefore worth reporting.

## Case presentation

A 10-year-old boy from the Indian subcontinent presented with persistent jaundice, intermittent itching, and the passage of clay-colored stool from 10 days after birth. His parents noticed a slowly growing lump over the upper part of his abdomen of 7 years’ duration. He also had a 6-year history of intermittent productive cough associated with sneezing, soreness of throat, rhinorrhea, and occasional fever. Episodes of productive cough usually occurred at intervals of 5–6 months (mostly during the winter months and rainy seasons), lasted for 3–5 days, and improved following treatment with expectorant and anti-allergic cold syrup. For the last 4 years he had experienced intermittent bleeding from his nose and the occasional appearance of post-traumatic violaceous-red patches at the site of trauma, as well recurrent post-traumatic fractures of his upper limbs following mild trauma. For the last 3 years, he had difficulty with night vision.

Our patient had no prior history of altered sensorium, blood vomiting, or passage of black stool. He was born 2 weeks after the expected date of delivery, had low birth weight (2.1 kg), delayed cry (cried after 5 minutes of birth), delayed mile stone development, and poor weight gain since early infancy. There was no family history of a similar type of illness, liver disorder, or consanguineous marriage: his parents, and his grandparents, were not related. He had no family or personal history of tuberculosis or, asthma. He had two elder asymptomatic healthy siblings.

**Fig. 1:**
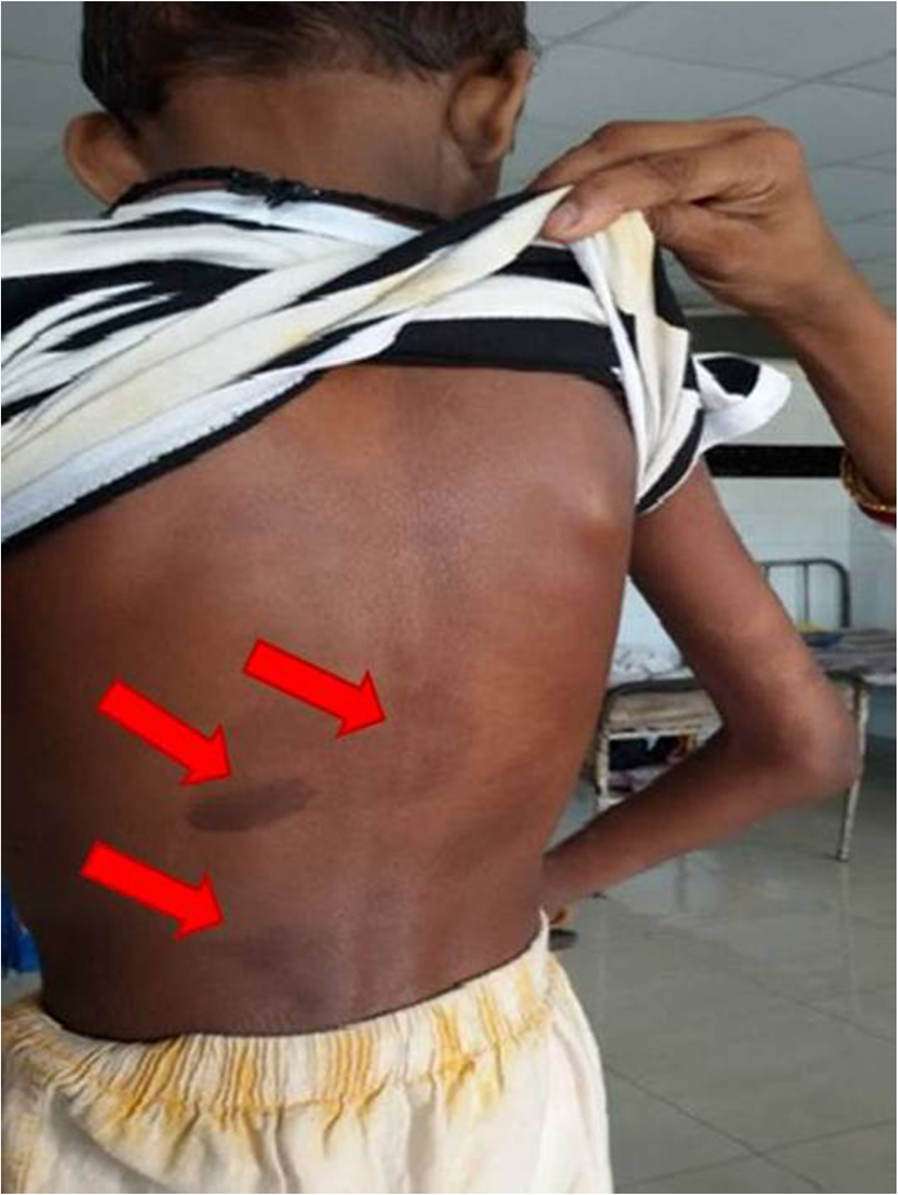
Skin manifestations of the patient. *Red arrows* indicate hyperpigmented patches

His height was 113 cm (z-score < −3), weight 13 kg (z-score < −3), body mass index 10.23 kg/m^2^, and mid-arm circumference 9 cm. He had mild pallor, icterus, clubbing, multiple hyperpigmented spots (café au lait spot), and scratch marks over his skin. There was no pedal edema, purpura, palmer erythema, spider nevi, gynecomastia, or testicular atrophy. Our patient had peculiar facial features in the form of a triangular face with broad forehead, deeply set eyes, hypertelorism, prominent ears, small pointed chin, and saddle nose with a bulbous tip. The skin manifestation is shown in Fig. 1, which illustrates the presence of multiple hyperpigmented spots resembling café au lait spots.

**Fig. 2:**
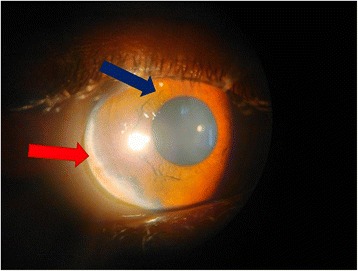
Ophthalmological findings of the patient. *Blue arrow* indicates an iris strand; *red arrow* indicates posterior embryotoxon

Examination of his abdomen revealed enlargement of the left lobe of his liver. His liver was firm in consistency with an irregular margin and slightly nodular surface. His liver span was 9 cm along the right mid-clavicular line and his spleen was palpable 3 cm below the left costal margin and firm in consistency. An eye examination demonstrated posterior embryotoxon, iris strands, increased intraocular pressure (Fig. 2). Findings from respiratory and neurological examinations were normal.

**Fig. 3:**
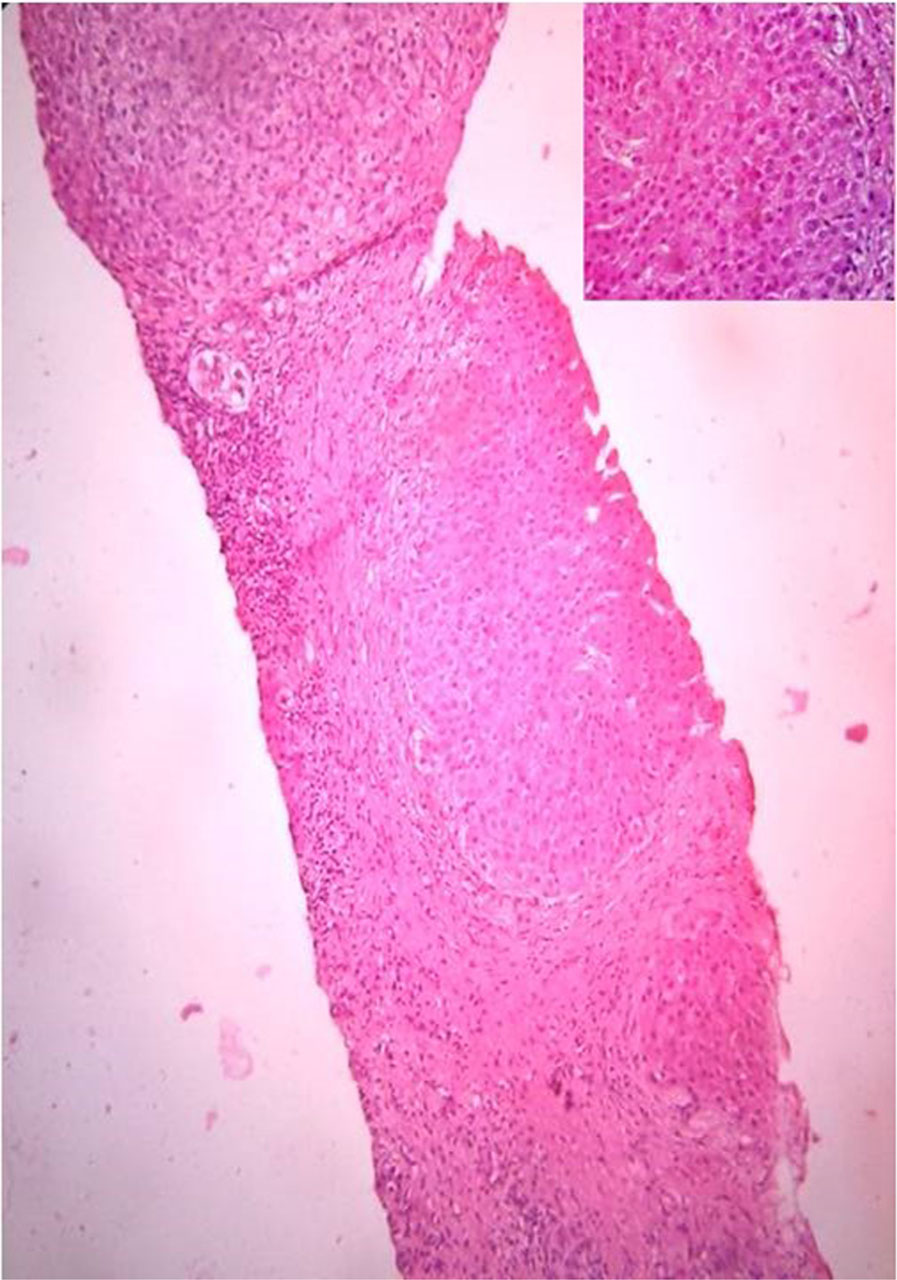
Liver histology findings of the patient

The biochemical parameters of the patient along with corresponding reference ranges for the laboratory are described in Table 1. He tested negative for hepatitis-B surface antigen and anti-hepatitis C virus antibodies. An X-ray of his wrist showed gross osteopenia, and an X- ray of his chest revealed cardiomegaly. Two-dimensional echocardiography showed pulmonary hypertension and tricuspid regurgitation. On gastroduodenoscopy there were no esophageal or gastric varices. An ultrasonographic study of his abdomen reported a shrunken right lobe with coarse hepatic parenchyma, an enlarged left lobe, mild splenomegaly, and poorly visualized hepatic and portal veins. Liver biopsy tissue stained with hematoxylin and eosin showed 10 portal tracts with a conspicuous absence of interlobular bile ducts adjacent to the hepatocytes, a portal area with mild inflammation, some degree of fibrosis, occasional nodules, and a few feathery hepatocytes without any inflammation (Fig. 3).

**Table 1:** The laboratory parameters of the patient along with corresponding reference ranges

Parameters	Values in the patient	Normal reference range	Inference
Hemoglobin	10.7 g/dL	14–16 g/dL	Decreased
TLC	8000/mm^3^	4000–11000/mm^3^	Normal
TPC	1.3 lakhs/mm^3^	1.5–4 lakhs/mm^3^	Decreased
INR (PT)	1.3	<1.2	Increased
Serum urea	18 mg/dL	15–45 mg/dL	Normal
Serum creatinine	0.7 mg/dL	0.5–1.5 mg/dL	Normal
Serum total bilirubin	4.1 mg/dL	0.1–1.2 mg/dL	Increased
Serum direct bilirubin	3.4 mg/dL	0.1–0.3 mg/dL	Increased
Serum AST	292 IU/L	10–40 IU/L	Increased
Serum ALT	121 IU/L	10–50 IU/L	Increased
Serum ALP	978 IU/L	250–750 IU/L	Increased
Serum GGT	24 IU/L	0–30 IU/L	Normal
Serum protein	5.8 g/dL	5.5–7.5 g/dL	Normal
Serum albumin	2.4 g/dL	3.5–5.5 g/dL	Decreased
Serum globulin	3.4 g/dL	2–3.5 g/dL	Normal
Serum calcium	8.6 mg/dL	9–10.5 g/dL	Decreased

*TLC* total leukocyte count, *TPC* total platelet count, *INR (PT)* international normalized ratio (prothrombin time), *AST* aspartate transaminase, *ALT* alanine transaminase, *ALP* alkaline phosphatase, *GGT* gamma glutamyl transpeptidase

Our patient was finally diagnosed as having the syndromic form of paucity of bile ducts (AGS) because, along with bile duct paucity, he had cholestatic jaundice and the typical ophthalmic and facial appearance. He was treated with ursodeoxycholic acid, vitamin D_3_, calcium, vitamin A solution, and multivitamins. His parents were advised to attend regular follow-up appointments but did not attend. Our patient died 1 year after the final diagnosis because of massive blood vomiting.

## Discussion

AGS (arteriohepatic dysplasia) is a rare disease with an incidence of 1 in 100,000 live births [6]. Over 500 cases of AGS have been reported since its initial description [1, 7]. AGS is diagnosed if three or more of the following five major features are present: cardiac murmur, posterior embryotoxon, butterfly-like vertebrae, renal abnormalities, and characteristic facies, along with histological diagnosis of bile duct paucity [8]. The exact prevalence of this syndrome in the Indian subcontinent is not known; only five cases have been reported so far [5, 9–13]. This is an autosomal dominant syndrome, with the gene (*JAG1*) being traced to chromosome 20 [13]. Mild to moderate mental retardation may be present. Patients commonly present before 6 months of age with either neonatal jaundice or cardiac murmurs; later they may present with poor linear growth, a broad forehead, pointed chin, deep-set eyes, and elongated nose with a bulbous tip. Hepatic disease is the predominant disease in AGS.

Our case had three out of the five major features of the syndrome with no vertebral or renal defects. He had cholestatic jaundice, typical facial features consistent with AGS, posterior embryotoxon, iris strands in the eye, and bile duct paucity demonstrated by histologic study. A “partial” or “incomplete” form of AGS was previously reported from India by Shendge et al. [11]. Our patient had an intermittent productive cough associated with rhinorrhea, soreness of throat, and sneezing mostly in the rainy seasons and winter months, which suggests the possibility of decreased immunity and recurrent viral infections.

The differential diagnoses of our case included progressive familial intrahepatic cholestasis (PFIC), childhood primary sclerosing cholangitis (PSC), congenital hepatic fibrosis, childhood autoimmune hepatitis, childhood primary biliary cirrhosis, alpha-1-antitrypsin deficiency, and cystic fibrosis.

PFIC is a clinical syndrome of intrahepatic cholestasis which presents in infancy or early childhood, and usually progresses rapidly to fibrosis and end-stage liver disease, whose confirmatory diagnosis can be made only following genetic analysis [14, 15]. Patients with PFIC can present with deafness, pancreatic insufficiency, cholelithiasis, and diarrhea, and it usually runs in families, which was not so in our case, ruling out the possibility of PFIC. Patients with PSC usually present with recurrent cholangitis and diarrhea because most patients usually have inflammatory bowel disease, especially ulcerative colitis [14, 15]. Our patient did not have such a presentation. Congenital hepatic fibrosis can present with upper gastrointestinal hemorrhage or recurrent cholangitis and can be associated with medullary sponge kidneys; these features were not present in our case. Diagnoses of childhood autoimmune hepatitis and childhood primary biliary cirrhosis can be confirmed based on typical liver histology findings and the presence of typical autoantibodies [14], which were absent in our case. Patients with alpha-1-antitrypsin deficiency can present with panacinar emphysema and can be diagnosed by typical liver histology finding such as the presence of periodic acid Schiff-positive diastase-resistant globules in the periportal hepatocytes, which were absent in our case. Patients with cystic fibrosis can present with meconium ileus, micro-gall bladder, and respiratory abnormalities; these features were absent in our case.

Having excluded these possibilities, our patient was finally diagnosed with AGS, most importantly because his symptoms fulfilled three out of the five criteria along with bile duct paucity in the liver histology, which is necessary for diagnosis of AGS. Our case was different from other previously published studies because of a history suggestive of fat-soluble vitamin deficiencies, such as a recurrent history of fracture in his upper limbs, night blindness, and recurrent epistaxis; he also had café au lait spots and very early onset of liver cirrhosis compared to previously published reports on AGS. Our case was quite different from other published case reports and we believe it may be the first case report on AGS in which the patient has café au lait spots as a skin manifestation.

Previous case reports of AGS with prominent skin manifestations have described the presence of well-defined, painless, indurated papules and plaques on the skin over the metacarpophalangeal and interphalangeal joints of the hands, eyelids, and the axillary, antecubital, inguinal, and popliteal folds of both sides, along with the presence of cutaneous xanthoma or tendinous xanthoma [9].

The long-term prognosis of AGS is uncertain, with congenital heart disease, hepatic cirrhosis, intracranial bleeding, and renal abnormalities being the commonest factors affecting mortality. Though a biliary diversion procedure can be undertaken to treat this disease, liver transplantation is the surgical treatment of choice. The estimated 20-year survival rates are 80% for those not requiring liver transplant and 60% for those requiring it [16]. Thus AGS is a rare and grave systemic disorder that should be part of the differential diagnosis of every case of prolonged neonatal jaundice and warrants aggressive treatment to minimize poor outcome.

### Limitations

Our case had certain limitations; we were not able to carry out the necessary genetic or chromosomal analysis for confirmation of the diagnosis of AGS. We were unable to carry out any genetic tests in our center because of the limited availability of sophisticated diagnostic facilities to diagnose any type of genetic disorder in our resource-constrained setting. Although our patient had an intermittent productive cough for 6 years, we were unable to perform sputum culture, a polymerase chain reaction test for virus infection, or screening to test for tuberculosis. We were also unable to perform assays of all the fat-soluble vitamins and other coagulation parameters except for total platelet count and international normalized ratio (prothrombin time) because of the limited diagnostic facilities in our resource-constrained setting.

## Conclusions

This particular syndromic form of paucity of bile duct disorder has been rarely reported in Indian literature so far. This is probably the first case report on AGS manifesting with café au lait syndrome and such an early onset of chronic liver disease, and therefore worth reporting. Because these patients usually have multisystem involvement, they may be evaluated by multiple clinical subspecialties and therefore it is necessary that all clinicians should be aware of this grave and rare disease, and they should take adequate measures for timely referral to the appropriate higher specialties so that a poor outcome can be prevented by early aggressive management.
